# Botulinum Toxin Therapy for Diseases in the Oral and Maxillofacial Regions: Current Evidence and Future Perspectives

**DOI:** 10.3390/toxins18090404

**Published:** 2026-09-21

**Authors:** Kazuya Yoshida

**Affiliations:** Department of Oral and Maxillofacial Surgery, National Hospital Organization Kyoto Medical Center, 1-1 Mukaihata-cho, Fukakusa, Fushimi-ku, Kyoto 612-8555, Japan; omdystonia@gmail.com

Botulinum neurotoxin (BoNT) therapy has developed from a treatment primarily used for neurological disorders into a versatile therapeutic modality applied across a broad range of medical and dental conditions [1,2]. The oral and maxillofacial region is particularly relevant to BoNT therapy because numerous functionally specialized muscles, sensory structures, temporomandibular joints, and salivary and autonomic structures are concentrated within a relatively small anatomical area. Disorders affecting these structures may impair mastication, swallowing, speech, facial expression, and quality of life. Conversely, inappropriate selection of target structures or excessive doses may interfere with these essential functions. Accurate diagnosis, detailed anatomical knowledge, appropriate target selection, and careful dosing are therefore fundamental to safe and effective treatment.

This Special Issue, entitled “Botulinum Toxin Therapy for Diseases in the Oral and Maxillofacial Regions”, brings together 11 contributions covering movement disorders, bruxism, temporomandibular disorders (TMDs), orofacial pain, masseter muscle hypertrophy, migraine, and basic mechanisms of BoNT action. Collectively, these articles illustrate the breadth of BoNT applications in the oral and maxillofacial region and the importance of multidisciplinary collaboration in their further development.

Oromandibular dystonia (OMD) is an important neurological indication for BoNT therapy in the stomatognathic system. Yoshida and Kaji (Contribution 1) conducted a systematic review and meta-analysis of onabotulinumtoxinA for OMD, including 26 studies and 1103 patients. Favorable responses were reported in 96.2% of patients, responses greater than 50% in 88.9%, and adverse effects in 17.8%, with dysphagia being the most common. These findings support the effectiveness of onabotulinumtoxinA for OMD while highlighting the need for randomized controlled trials and more standardized treatment and outcome assessment.

Ferrillo et al. (Contribution 2) reviewed the use of BoNT/A for masseter muscle hypertrophy, focusing on its mechanisms, injection protocols, clinical effects, and adverse events. BoNT/A is an established nonsurgical option for reducing masseter volume and lower-face width, but there is still no consensus regarding the optimal dose or injection protocol. Reported adverse effects are generally mild and transient, although accurate injection within the masseter is essential to minimize unwanted functional and esthetic effects. The review emphasizes the need for more standardized treatment protocols.

Park et al. (Contribution 3) examined changes in masticatory muscle thickness and pain after BoNT-A injections in 15 patients with myogenic TMD. Ultrasonography showed significant thinning of the masseter and temporalis muscles within 2 weeks, persisting for 12 weeks. Pain intensity also tended to decrease, particularly in the early period, but the reductions were not consistently significant and showed no persistent correlation with muscle thickness. These findings suggest that pain relief after BoNT-A cannot be explained solely by muscle atrophy.

Furuhata et al. (Contribution 4) evaluated 304 patients with bruxism treated with bilateral masseter BoNT injections. Masseter electromyographic activity and the maximal bite force decreased significantly, while maximal mouth opening, sleep quality, shoulder and neck stiffness, and headaches improved. Age, sex, and baseline muscle activity or bite force influenced the magnitude of treatment effects. The study therefore supports individualized dose adjustment rather than a uniform dosing strategy.

Matusz et al. (Contribution 5) performed the first longitudinal study using myotonometry to quantify masseter muscle changes after BoNT-A treatment in 57 patients with bruxism-related symptoms. Significant changes were observed in muscle tone, stiffness, elasticity, relaxation time, and creep, with the greatest and most sustained effects in patients with higher baseline muscle values. The study supports myotonometry as an objective tool for monitoring masticatory muscle function, while also indicating that randomized controlled trials are needed to confirm clinical efficacy.

Argyriou et al. (Contribution 6) prospectively studied 58 patients with chronic migraine and comorbid bruxism who received two consecutive onabotulinumtoxinA cycles. Treatment significantly reduced migraine frequency, disability, bruxism-related pain, and masseter hypertrophy, with 70.7% of patients achieving at least a 50% reduction in monthly headache days. Improvement in bruxism-related pain was strongly correlated with the reduction in headache frequency. These real-world findings suggest that onabotulinumtoxinA may provide concurrent benefits for chronic migraine and comorbid bruxism.

Sharav et al. (Contribution 7) proposed a unifying hypothesis for the analgesic effects of BoNT-A across neuropathic, myofascial, and neurovascular orofacial pain conditions. They suggest two complementary mechanisms: segmental neural blockade and retrograde axonal transport leading to central neuromodulation. This framework integrates clinical and preclinical observations and may help explain why BoNT-A can reduce pain across disorders with different etiologies. The hypothesis also provides a basis for future mechanism-oriented studies of orofacial pain therapy.

Fabillar et al. (Contribution 8) reviewed experimental animal studies examining the antinociceptive mechanisms of BoNT/A in orofacial pain. The proposed mechanisms include retrograde transport, regulation of neuronal excitability, inhibition of neuropeptide release, modulation of inflammation, and stimulation of opioid systems in both peripheral and central pathways. These findings support a multidimensional action of BoNT/A beyond neuromuscular blockade. Further studies are required to determine how these experimental mechanisms translate into clinical pain treatment.

Chaudhry et al. (Contribution 9) reviewed peripheral and central sensitization in TMDs and the potential mechanisms of BoNT-A therapy. Preclinical and clinical evidence suggests that BoNT-A can reduce peripheral neurotransmitter release, neurogenic inflammation, and central neuronal excitability, although clinical analgesic effects remain modest and variable. Patients with sensory sensitization may be more likely to respond, but methodological heterogeneity limits firm conclusions. The authors therefore propose BoNT-A as a potential adjunctive, mechanism-based treatment within multimodal TMD management and emphasize the need for standardized sensory phenotyping, dosing, and safety monitoring.

Papetti et al. (Contribution 10) reviewed current evidence for onabotulinumtoxinA in children and adolescents with chronic migraine. Although the treatment is approved for the prevention of chronic migraine in adults, pediatric use remains off-label and the evidence is still limited. This review summarizes randomized trials, prospective studies, and retrospective series addressing efficacy, safety, tolerability, and practical clinical considerations. Larger controlled studies and long-term follow-up are needed before standardized pediatric treatment protocols can be established.

Finally, Al-Sabi and Lawrence (Contribution 11) investigated synaptic plasticity after BoNT/A-induced blockade of mouse submandibular parasympathetic ganglia. Paralysis was associated with a rapid and marked, but ultimately reversible, reduction in presynaptic vesicle proteins, SNAP-25, and postsynaptic nicotinic acetylcholine receptors. These findings provide insight into the biochemical remodeling of autonomic synapses after BoNT/A exposure. They may also help explain why BoNT/A-induced autonomic paralysis can persist longer than muscle weakness, which is relevant to salivary and other autonomic applications of BoNT therapy.

Taken together, the contributions to this Special Issue demonstrate that the oral and maxillofacial region represents a broad and promising field for BoNT therapy. Importantly, BoNT should not be regarded simply as a means of weakening overactive muscles. Its therapeutic actions may encompass modulation of abnormal motor activity, peripheral and central pain processing, neurogenic inflammation, autonomic neurotransmission, and muscle biomechanical properties. At the same time, the diversity of the disorders discussed in this Special Issue makes it clear that BoNT should not be used as a nonspecific treatment for symptoms such as pain, clenching, muscle tenderness, or increased muscle activity. Accurate diagnosis must precede treatment.

The oral and maxillofacial region also poses distinctive therapeutic challenges because many target muscles participate in essential functions including mastication, swallowing, speech, and facial expression. Treatment should therefore be diagnosis-driven, target-specific, dose-conscious, and function-preserving. Detailed knowledge of anatomy, appropriate use of electromyographic or ultrasonographic guidance when necessary, and objective evaluation of treatment effects can further improve the precision and safety of BoNT therapy [3].

Another central message emerging from this Special Issue is the importance of collaboration between medicine and dentistry. Dentists and oral and maxillofacial surgeons routinely evaluate mastication, occlusion, temporomandibular joint function, oral movements, and other functions of the stomatognathic system and possess detailed anatomical expertise in this region [3]. At the same time, many of the conditions considered here—including OMD, chronic pain, migraine, and other neurological disorders—cross conventional specialty boundaries. Close cooperation among dentistry, oral and maxillofacial surgery, neurology, pain medicine, otorhinolaryngology, rehabilitation medicine, and other disciplines is therefore essential for accurate diagnosis, appropriate patient selection, and safe treatment. This multidisciplinary concept was also a central aim of this Special Issue.

Despite substantial advances, several important challenges remain. Future research should prioritize precise clinical phenotyping, standardized diagnostic criteria, optimization of target selection, injection sites and doses, objective and patient-reported outcome measures, systematic assessment of adverse events, and long-term safety. Most importantly, prospective multicenter controlled studies and multidisciplinary clinical trials will be necessary to establish higher-level evidence. Such evidence is essential not only to improve clinical practice but also to facilitate regulatory approval for indications that currently remain off-label in many countries.

This Special Issue illustrates both the current achievements and the future potential of BoNT therapy in the oral and maxillofacial region. The convergence of detailed anatomical knowledge, increasingly precise injection techniques, growing understanding of neurophysiological and analgesic mechanisms, and multidisciplinary clinical collaboration provides a strong foundation for further development of this field. We sincerely thank all authors for their valuable contributions, the reviewers for their careful assessments and constructive comments, and the editorial staff of *Toxins* for their continued support in bringing this Special Issue to completion. We hope that this collection will stimulate further interdisciplinary research and contribute to safer, more precise, and evidence-based BoNT therapy for patients with oral and maxillofacial disorders.
